# Variations of Wnt/β‐catenin pathway‐related genes in susceptibility to knee osteoarthritis: A three‐centre case‐control study

**DOI:** 10.1111/jcmm.14696

**Published:** 2019-09-27

**Authors:** Yong Huang, Lifeng Jiang, Haoyu Yang, Lidong Wu, Nanwei Xu, Xindie Zhou, Jin Li

**Affiliations:** ^1^ Department of Orthopedics The Affiliated Changzhou No. 2 People’s Hospital of Nanjing Medical University Changzhou China; ^2^ Department of Orthopedic Surgery The Second Affiliated Hospital Zhejiang University School of Medicine Hangzhou China; ^3^ Department of Orthopedic Surgery The Second Affiliated Hospital of Jiaxing University Jiaxing China

**Keywords:** bioinformatics analysis, genetic risk score, knee osteoarthritis, polymorphism, Wnt

## Abstract

Excessive activation of the Wnt signalling pathway in the articular cartilage is demonstrated to be related to the onset and severity of osteoarthritis (OA). However, few studies have investigated the association between variants in Wnt‐pathway‐related genes and the risk of OA by searching Pubmed and EMBASE. Totally, 471 knee OA patients and 532 controls were recruited from three hospitals to evaluate the associations of five genetic variants (rs61735963, rs2908004, rs10795550, rs1799986 and rs1127379) with the risk of knee OA. These polymorphisms were genotyped through polymerase chain reaction and Sanger sequencing. Genetic risk scores (GRSs) were calculated to evaluate the combined effect of these genetic variants. No significant association was found between OA risk and polymorphisms (rs61735963, rs10795550 or rs1127379). However, WNT16 rs2908004 polymorphism was correlated with a decreased risk of OA, especially among females, smokers, non‐drinkers and individuals with age < 60 years or BMI ≥ 25. This SNP was also associated with Kellgren‐Lawrence grade and CRP. Similarly, LRP1 rs1799986 polymorphism decreased the risk of OA among males, smokers, drinkers and individuals with age < 60 years or BMI ≥ 25. TT genotype was more frequent in the group of VAS ≥ 6 versus VAS < 6. A low GRS was positively correlated with a decreased risk of OA. In addition, rs2908004 or rs1799986 polymorphism reduces the expression of WNT16 or LRP1. In conclusion, two SNPs (rs2908004 and rs1799986) are associated with the decreased risk of OA by regulating the Wnt pathway.

## 1 INTRODUCTION

Osteoarthritis (OA) is the most prevalent chronic joint disease that contributes to cartilage matrix degradation.^1^ Major mechanism of OA is characterized by abnormal joint tissue metabolism and results in cartilage degradation, bone remodelling, joint inflammation, osteophyte formation and loss of normal joint function.^2^ About 9.6% of men and 18% of women ≥ 60 years old suffer from symptomatic OA worldwide.^3^ Multiple factors, including advanced age, excessive body weight, repeated trauma or surgery to the joint structures, diabetes and hormone‐related disorders, contribute to increased risk of OA.^4^ Studies provide clear evidence of a heritable component in susceptibility of OA,^2^, ^5^, ^6^ but the specific genetic factors are largely unknown. It therefore remains a challenge to identify risk alleles or candidate genes that contribute to OA pathogenesis.

The Wnt/β‐catenin signalling pathway plays a crucial role in the regulation, proliferation, differentiation and cellular death processes in numerous anomalies of development, growth and homeostasis in animal organisms.^7^ We previously found the Wnt/β‐catenin pathway plays a key role in OA by inducing the expression of matrix metalloproteinases.^8^ Besides, it contributes to cell differentiation and apoptosis and directly regulates chondrocyte phenotype.^9^, ^10^ Once Wnt protein is secreted, it binds to the frizzled receptor and low‐density lipoprotein receptor‐related protein 5/6 (LRP5/6) in order to stabilize the β‐catenin protein and initiate an intricate signalling cascade.^11^

Abnormal activation of the Wnt/β‐catenin signalling is involved in the OA pathology, but little is known about the association of the Wnt pathway‐related gene polymorphisms with OA susceptibility. Thus, this hospital‐based case‐control study was conducted to investigate the effects of Wnt pathway‐related genes (DVL1, WNT16, ITIH5, LRP1 and SFRP1) polymorphisms on the risk of OA in a Chinese population.

## 2 MATERIALS AND METHODS

### 2.1 Search strategy

We systematically searched the Pubmed and EMBASE databases up to 12 January 2019 to identify eligible studies using the following keywords: ‘osteoarthritis’, ‘OA’, ‘wnt signalling pathway’, ‘Wnt/β‐catenin signalling pathway’, ‘polymorphism’, ‘SNP’ and ‘variant’. We also checked references in eligible studies and reviews for other relevant studies. No restriction was set on language or ethnicity.

### 2.2 Protein‐protein interaction network and functional annotation

We constructed protein‐protein interaction (PPI) network to identify significant interactions among proteins encoded by Wnt pathway‐related genes using the search tool STRING (http://string-db.org/).^12^ PPI network was constructed by the PPI pairs with protein interaction scores exceeding 0.4. Gene Ontology (GO) is a widely adopted source of gene functional annotation, including biological process, molecular function and cellular component.^13^

### 2.3 Study population

We recruited 471 knee OA patients and 532 healthy controls from the Affiliated Changzhou No. 2 People's Hospital of Nanjing Medical University, the Second Affiliated Hospital of Jiaxing University and the Second Affiliated Hospital, Zhejiang University School of Medicine between November 2014 and July 2018. Knee OA was diagnosed in accordance with the clinical and radiographic criteria of the American College of Rheumatology (1987).^14^ Exclusion criteria for OA patients were as follows: (a) autoimmune disorders, malignant tumour, cardiovascular diseases and liver and kidney dysfunction; (b) history of arthritis; and (c) drug abuse more than 3 months. The healthy controls were selected from the outpatients of the above hospitals during the same period. X‐ray analysis confirmed no evident symptoms of joint pain and the absence of knee OA. Demographic and clinical information (C‐reactive protein (CRP), erythrocyte sedimentation rate (ESR), visual analogue score (VAS) and Kellgren‐Lawrence grade) was collected from all participants by using a written questionnaire and reviewing medical records. Individuals who smoked at least one cigarette daily were considered as smokers. Alcohol usage was defined as consumption of at least three alcoholic drinks one week. This study was approved by the Ethics Committees of the above three hospitals and was consistent with the Declaration of Helsinki. Each subject provided written informed consent prior to participation.

### 2.4 SNP selection process

Selection process was as follows. First, we identified five OA‐related genes (DVL1, WNT16, ITIH5, LRP1 and SFRP1) on the Wnt signalling pathway by reviewing the literature. Second, we found out whether the SNPs on these genes have been studied in the disease. Third, SNPs with minor allele frequency > 0.5, located in the promoter, exon or 3’‐UTR were selected. In total, five SNPs in Wnt signalling pathway‐related genes were selected, including rs61735963, rs2908004, rs10795550, rs1799986 and rs1127379.

### 2.5 Genotyping

Peripheral blood (2 mL) was collected from each subject. Genomic DNA was extracted using a QIAamp DNA blood mini kit (Qiagen, Hilden, Germany) according to the manufacturer's instruction. DNA samples were tested for purity and concentration with an ultraviolet spectrophotometer and stored at −20°C. PCR primers were designed on Primer Premier 5.0 (Premier, Pala Alto, CA, USA). PCR amplification was conducted in 25‐μL volumes each containing 1 μL of the DNA template, 2.5 μL of 3 mmol/L dNTPs, 1.0 μL of each primer (10 mmol/L), 2.5 μL of 10× PCR buffer (added with MgCl_2_), 1.0 U of Taq DNA polymerase and ddH_2_O. DNA was denatured at 95°C for 6 minutes, then amplified by 35 PCR cycles (95°C for 30 seconds, 50°C for 1 minutes, 72°C for 30 seconds) and finally extended at 72°C for 10 minutes. PCR products were revealed on 2% agarose gel electrophoresis with ethidium bromide, purified using a DNA purification kit and then genotyped through Sanger sequencing.

### 2.6 Reverse transcription‐polymerase chain reaction

Total RNA was isolated from the whole blood from the OA patients and healthy controls using the Trizol reagent (Invitrogen, Carlsbad, CA, USA) following the manufacturer's instructions. Five genes (DVL1, WNT16, ITIH5, LRP1 and SFRP1) expression were detected by RT‐PCR with Hifair^TM^ first‐strand cDNA synthesis SuperMix and qPCR SYBR Green Master Mix (Yesen, Shanghai, China). Forward and reverse primers used for PCR were as follows: 5'‐CGTAAAGTGCTGGGGACCTT‐3', 5'‐GATCCACGCACTCAGGAACT‐3' (SFRP1); 5'‐TCCTCACTAACCAGCTCCGT‐3', 5'‐GCGCTCATGTCACTCTTCAC‐3' (DVL1); 5'‐CTACAGCTCCCTGCAAACGA‐3', 5'‐AGCACCAAGTTATCCCTCGC‐3' (WNT16); 5'‐GTCCCTGTGTGTGGGGTC‐3', 5'‐ACCCTGAGTCCATCCTGCTC‐3' (ITIH5); 5'‐CCGCTCTGATGAGTCTGCTT‐3', 5'‐AGTCATTGTCATTGTCGCATCT‐3' (LRP1); 5'‐GATGAGATTGGCATGGCTTT‐3', 5'‐GTCACCTTCACCGTTCCAGT‐3' (β‐actin). Statistical analysis used relative quantification, and the 2^−△△CT^ method was used to calculate relative expression.

### 2.7 Statistical analysis

Continuous variables were expressed as mean ± standard deviation and analysed using Student's *t* test or Mann‐Whitney *U* test. Categorical variables were reported as frequency and percentage and analysed using chi‐squared test. The genotype frequencies among controls in line with Hardy‐Weinberg equilibrium (HWE) were investigated through goodness‐of‐fit chi‐squared test. The risk of gene for OA was evaluated by calculating odds ratios (ORs) and 95% confidence intervals (CIs). Stratification analysis was conducted according to sex, age, smoking, alcohol consumption and body mass index (BMI). The effect size of each SNP was investigated using crude ORs. If the SNPs’ disease risk was independent, the weighted genetic risk score (GRS) was estimated as the sum of (log OR of SNP) × (number of risk alleles carried by the individual) across five SNPs. The GRS was then divided into quartiles according to the normalized value and calculated by binary logistic regression. All statistical analyses were performed on SAS 9.1.3 (SAS Institute, Cary, NC, USA). *P* < .05 was considered statistically significant.

## 3 RESULTS

### 3.1 Study characteristics

The systematic database searching returned 25 articles, of which 12 articles were excluded, including three duplicates, and nine reviews, meeting reports or unrelated articles. Finally, 13 articles involving 20 genes and 127 SNPs were included for further analysis. The detailed characteristics of the selected studies are presented in Table 1, including 6 case‐control studies, four case‐only studies and three cohort studies. Ten articles were conducted among Caucasians and three among Asians. Twelve of the 127 SNPs were associated with the risk of OA (Table 2). Most of the SNPs were located at the intron region of genes. Seven SNPs (rs12341259, rs851054, rs986690, rs41494349, rs7164503, rs12593365 and rs2908004) decreased and five SNPs (rs2353525, rs409238, rs2615977, rs2707466 and rs2929970) increased the risk of OA.

**Table 1:** Characteristics of included studies

Author and year	Type of study	Country	Sample size	Genotyping method	Polymorphism	QAS
Loughlin2004	Family case‐control	UK	OA/control 936/760	PCR‐RFLP	TNFAIP6 rs1046668; ITGA6 rs2737085 rs10209072; FRZB rs288326 rs7775	8
Urano2007	Case‐only	Japan	Spinal OA 304	TaqMan	W1SP1 2364A/G	7
Kerkhof2008	Cohort	Netherlands	Hip/knee/hand OA 2507	TaqMan	FRZB rs288326 rs7775; LRP5 Ala1330val; LRP6 lle1062val	8
Urano2008	Case‐only	Japan	Spinal OA 357	TaqMan	LRP5 Q89R	7
Valdes2009	Case‐control	UK	Knee/hip OA/control 2170/2849	PCR	ANP32A rs7164503	7
Lamas2010	Case‐control	Spain	OA/control 191/283	N/A	COL10A1 rs11965969	7
Gao2010	Family cohort	China	Osteoarthritis 1260	TaqMan	FZRB rs643399 rs409238 rs288324 rs4666865	7
Leijten2012	Cohort	Netherlands	OA 7142	N/A	GREM1 rs1881537 rs921510 rs4779584 rs1258726 rs12593365 DKK1 rs11636374 rs12905294 rs16959110 rs16959022 rs12593223 rs11071936 rs10318	8
Baker‐LePain2012	Case‐control	USA	Hip OA/controls 451/601	PCR	FRZB (rs288326 rs7775)	9
Garca‐Ibarbia2013	Case‐only	Spain	Hip fractures/hip OA 500/353	Sequencing	WNT4 (rs7526484 rs2235526 rs10917158) WNT10A (rs3806557 rs10177996 rs2385199) WNT16 (rs3779381 rs2908004 rs2707471 rs3801385 rs2707466 rs17143305) SFRP1 (rs3242 rs1127379 rs7820647 rs11786592 rs6651363 rs10109536 rs17652488 rs10958671 rs17574424 rs7832767 rs968427 rs921142)	9
Garca‐Ibarbia2013	Case‐control	Spain	Hip/knee OA/control 680/606	TaqMan	WNT1 (rs11610885 rs10878228 rs7397906 rs6581609 rs1900292); WNT10A (rs3806557 rs10177996 rs2385199) WNT16 (rs3779381 rs29008004 rs2707471 rs3801385 rs2707466 rs17143305) DVL2 (rs2074222 rs222837 rs222836 rs2074216) FZD5 (rs3731567 rs35994626) BCL9 (rs666569 rs10793689 rs17160413 rs12081028 rs6659237 rs1546161 rs10793690 rs7523564 rs1015235 rs2353544 rs11240082 rs11240083 rs10900382 rs2353525 rs688325 rs6675828 rs3737843 rs11807878 rs3766512) SFRP1 (rs3242 rs1127379 rs7820647 rs11786592 rs6651363 rs10109536 rs17652488 rs10958671 rs17574424 rs7832767 rs968427 rs921142) SFRP4 (rs2598116 rs1052981 rs1802074 rs1802073 rs2598115 rs1450856 rs3807192) TCF7L1 (rs3810822 rs2568217 rs2568225 rs11682047 rs11693369 rs2568197 rs2568203 rs2568210 rs4832148 rs12477587 rs17763853 rs7581133 rs11904127 rs6547606 rs10204252 rs17764504 rs7574856 rs4832156 rs6741339 rs11547160	10
Garca‐Ibarbia2017	Case‐only	Spain	Hip OA/knee OA 173/52	TaqMan	WNT16 rs2707466 rs2908004	8
Fernandez‐Torres2018	Case‐control	Mexico	Knee OA/control 141/190	TaqMan	LRP6 rs12314259; SOST rs851054; FMN2 rs986690; FRZB rs409328; COL11A1 rs265977	8

**Table 2:** Positive sites on the Wnt signalling pathway‐related genes

SNP	Gene	Major/minor allele	MAF	Location	Results	Country
Rs2353525	BCL9	T/G	0.4293/1250	Intron	Risk	Spain
Rs12341259	LRP6	C/T	0.0094/47	Intron	Protect	Mexico
Rs851054	SOST	C/A	0.4441/2224	Intron/3’‐UTR	Protect	Mexico
Rs986690	FMN2	G/A	0.3203/1604	Intron	Protect	Mexico
Rs409238	FRZB	G/A	0.4301/2154	Intron	Risk	Mexico
Rs2615977	COL11A1	A/C	0.2194/1099	Intron	Risk	Mexico
Rs2707466	WNT16	C/T	0.4970/2489	Exon	Risk	Spain
Rs2929970	WISP1	G/A	0.4441/2224	3’‐UTR	Risk	Japan
Rs41494349	LRP5	A/G	0.0200/100	Exon/promoter	Protect	Japan
Rs7164503	ANP32A	T/C	0.3816/1911	Intron	Protect	UK
Rs12593365	GREM1	T/C	0.2165/1084	Exon	Protect	Netherlands
Rs2908004	WNT16	G/A	0.4896/2452	Exon	Protect	Spain

### 3.2 Bioinformatics analysis

**Figure 1:**
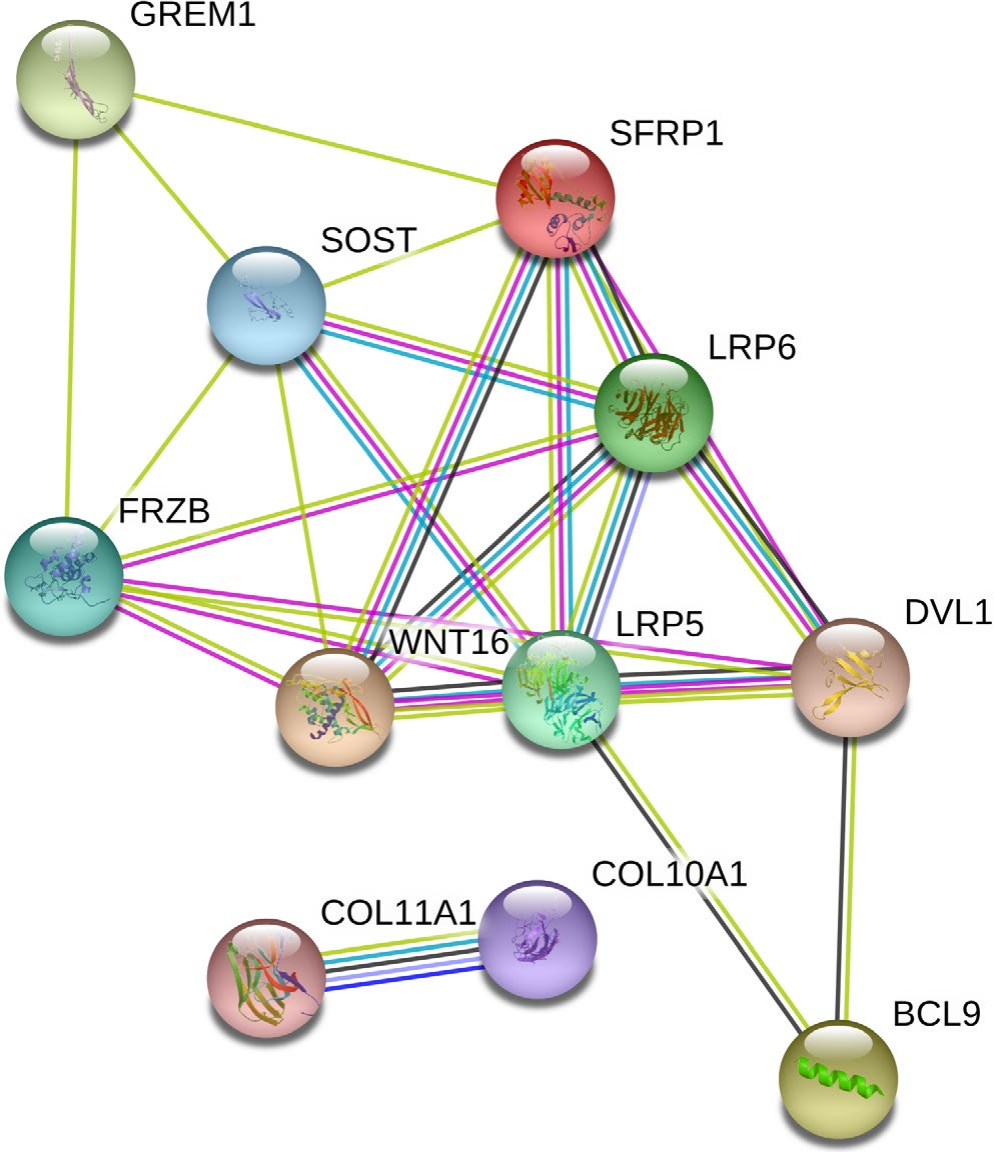
The protein interaction network of Wnt/β‐catenin pathway‐related genes

**Figure 2:**
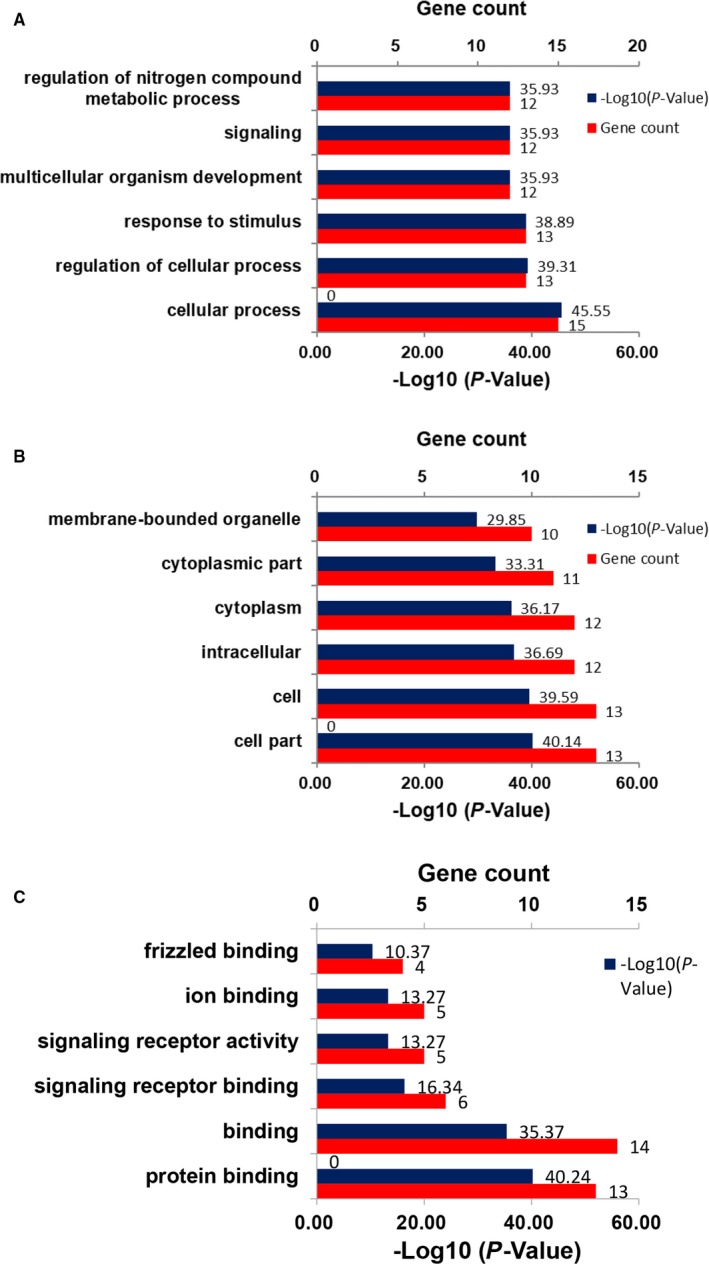
Barplot of representative GO analysis of Wnt/β‐catenin pathway‐related genes. A, Biological process; B, cellular component; C, molecular function

We constructed the PPI network of the Wnt pathway genes associated with OA reported in the literature (Figure 1). Three genes (WNT16, LRP5 and LRP6) were considered as key genes in the PPI module. GO analysis indicated these genes were significantly related to cellular process (biological process), cell part (cellular component) and protein binding (molecular function) (Figure 2).

### 3.3 Clinical parameters of the study population

The OA patients and controls were aged 57.87 and 57.47 years on average respectively (Table 3). About 54% of the participants in the two groups were females. The average BMIs did not differ significantly between groups (*P* = .632). The majority of OA patients were assigned with Kellgren‐Lawrence grade III + IV. Clinical characteristics (eg ESR, CRP, VAS and Lequesnes’ index) of patients were also collected to investigate the pathology of OA.

**Table 3:** Patient demographics and risk factors in osteoarthritis

Variable	Cases (n = 471)	Controls (n = 532)	*P*
Age (y)	57.87 ± 8.79	57.47 ± 9.25	.482
Sex			
Male	214 (45.4%)	248 (46.6%)	.708
Female	257 (54.6%)	284 (53.4%)	
BMI	25.04 ± 3.95	24.92 ± 3.78	.632
Smoking			
Yes	201 (42.7%)	244 (45.9%)	.310
No	270 (57.3%)	288 (54.1%)	
Alcohol			
Yes	238 (50.5%)	239 (44.9%)	.076
No	233 (49.5%)	293 (55.1%)	
ESR	11.52 ± 13.52	–	–
CRP	7.15 ± 15.67	–	–
VAS	7.92 ± 1.25	–	–
Lequesnes’ index	15.32 ± 1.88	–	–
L/R knee OA			
Left	252 (53.5%)	–	–
Right	219 (46.5%)	–	
Kellgren‐Lawrence grade			
II	65 (13.8%)	–	–
III	198 (42.0%)	–	
IV	208 (44.2%)	–	

Abbreviations: BMI, body mass index; CRP, C‐reactive protein; ESR, erythrocyte sedimentation rate; VAS, visual analogue scale.

### 3.4 Wnt pathway‐related genes variant analyses

The genotype and allele distributions of five SNPs are shown in Table 4. No significant deviation from HWE was found for any SNP in either group. WNT16 rs2908004 polymorphism was associated with a decreased risk of OA in the co‐dominant, recessive, dominant and allelic models. Additionally, the TT genotype versus CC genotype of rs1799986 polymorphism had a 0.49‐fold decreased risk of OA (TT vs. CC: OR 0.49; 95%CI 0.29‐0.83; *P* = .008). Furthermore, the CT + TT genotype significantly increased OA risk (CT + TT vs. CC: OR 0.72; 95%CI 0.56‐0.93; *P* = .011). Similar findings were observed in the dominant and allelic models. Notably, these associations were still significant after adjusting sex and age. No meaningfully or statistically significant associations with OA risk were noted in other polymorphisms.

**Table 4:** Logistic regression analysis of associations between polymorphisms and risk of osteoarthritis

Genotype	Cases^a^ (n = 471)		Controls^a^ (n = 532)		OR (95% CI)	*P*	OR (95% CI)^b^	*P* b
	n	%	n	%				
rs2908004 G/A								
GG	158	33.6	138	26.0	1.00	–	–	–
GA	228	48.5	265	49.9	0.75 (0.57‐1.01)	0.056	0.75 (0.57‐1.01)	0.056
AA	84	17.9	128	24.1	**0.57 (0.40‐0.82)**	**0.002**	**0.57 (0.40‐0.82)**	**0.002**
GA + AA	312	66.4	393	74.0	**0.70 (0.53‐0.91)**	**0.009**	**0.69 (0.53‐0.91)**	**0.009**
GG + GA	386	82.1	403	75.9	1.00	–	–	–
AA	84	17.9	128	24.1	**0.68 (0.50‐0.93)**	**0.016**	**0.68 (0.50‐0.93)**	**0.015**
G allele	544	57.9	541	50.9	1.00	–	–	–
A allele	396	42.1	521	49.1	**0.76 (0.63‐0.90)**	**0.002**	–	–
rs10795550 T/C								
TT	285	60.6	305	57.4	1.00	–	–	–
TC	162	34.5	195	36.7	0.89 (0.69‐1.16)	0.404	0.90 (0.69‐1.17)	0.418
CC	23	4.9	31	5.8	0.79 (0.45‐1.39)	0.422	0.79 (0.45‐1.39)	0.418
TC + CC	185	39.4	226	42.6	0.88 (0.68‐1.13)	0.322	0.88 (0.68‐1.14)	0.328
TT + TC	447	95.1	500	94.2	1.00	–	–	–
CC	23	4.9	31	5.8	0.83 (0.48‐1.44)	0.505	0.83 (0.47‐1.44)	0.498
T allele	732	77.9	805	75.8	1.00	–	–	–
C allele	208	22.1	257	24.2	0.89 (0.72‐1.10)	0.273	–	–
rs1127379 T/C								
TT	120	25.6	146	27.5	1.00	–	–	–
TC	234	49.9	265	49.9	1.07 (0.80‐1.45)	0.639	1.08 (0.80‐1.45)	0.624
CC	115	24.5	120	22.6	1.18 (0.83‐1.67)	0.368	1.18 (0.83‐1.68)	0.358
TC + CC	349	74.4	385	72.5	1.11 (0.84‐1.47)	0.485	1.11 (0.84‐1.47)	0.471
TT + TC	354	75.5	411	77.4	1.00			
CC	115	24.5	120	22.6	1.12 (0.84‐1.50)	0.441	1.12 (0.84‐1.51)	0.436
T allele	474	50.5	557	52.4	1.00	–	–	–
C allele	464	49.5	505	47.6	1.08 (0.91‐1.29)	0.392	–	–
rs61735963 C/T								
CC	403	85.7	444	83.6	1.00	–	–	–
CT	64	13.6	83	15.6	0.85 (0.60‐1.21)	0.359	0.85 (0.60‐1.21)	0.358
TT	3	0.6	4	0.8	0.82 (0.18‐3.71)	0.801	0.82 (0.18‐3.68)	0.793
CT + TT	67	14.3	87	16.4	0.85 (0.60‐1.20)	0.345	0.85 (0.60‐1.20)	0.348
CC + CT	467	99.4	527	99.2	1.00	–	–	–
TT	3	0.6	4	0.8	0.85 (0.19‐3.80)	0.827	0.84 (0.19‐3.77)	0.819
C allele	870	92.6	971	91.4	1.00	–	–	–
T allele	70	7.4	91	8.6	0.86 (0.62‐1.19)	0.357	–	–
rs1799986 C/T								
CC	287	61.1	282	53.1	1.00	–	–	–
CT	160	34.0	203	38.2	0.77 (0.59‐1.01)	0.055	0.77 (0.59‐1.01)	0.056
TT	23	4.9	46	8.7	**0.49 (0.29‐0.83)**	**0.008**	**0.49 (0.29‐0.83)**	**0.008**
CT + TT	183	38.9	249	46.9	**0.72 (0.56‐0.93)**	**0.011**	**0.72 (0.56‐0.93)**	**0.011**
CC + CT	447	95.1	485	91.3	1.00	–	–	–
TT	23	4.9	46	8.7	**0.54 (0.32‐0.91)**	**0.020**	**0.54 (0.32‐0.91)**	**0.021**
C allele	734	78.1	767	72.2	1.00	–	–	–
T allele	206	21.9	295	27.8	**0.73 (0.60‐0.90)**	**0.003**	–	–

Note

Bold values are statistically significant (*P* < .05).

a The genotyping was successful in 470 cases and 531 controls for rs2908004, rs10795550, rs61735963 and rs1799986; the genotyping was successful in 469 cases and 531 controls for rs1127379.

b Adjust for age and sex.

Stratified analyses by sex, age, smoking, drinking and BMI were conducted (Table 5). For rs2908004, the decreased effect was more evident in the subgroups of females, age < 60 and BMI ≥ 25. In addition, the decreased association between LRP1 rs1799986 polymorphism was stronger in the subgroups of males, smokers, non‐drinkers, age < 60 and BMI ≥ 25. Subsequently, we investigated the possible association between the two above polymorphisms and clinical characteristics (CRP, ESR, VAS, Lequesnes’ index and K‐L grade) of OA patients (Table 6). GG + AA genotype carriers of rs2908004 polymorphism had lower CRP expression and K‐L grade compared to the GG genotype. For rs1799986, the TT genotype was more frequent in the group of VAS ≥ 6 versus VAS < 6.

**Table 5:** Stratified analyses between rs2908004/rs1799986 polymorphisms and the risk of osteoarthritis

Variable	Case/control			Heterozygous model	Homozygous model	Recessive model	Dominant model
Rs2908004	GG	GA	AA	GA vs. GG	AA vs.GG	AA vs. GA + GG	AA + GA vs.GG
Sex							
Male	69/71	108/121	36/56	0.93 (0.61‐1.41); 0.721	0.66 (0.39‐1.13); 0.129	0.69 (0.44‐1.11); 0.124	0.84 (0.57‐1.25); 0.397
Female	89/67	120/144	48/72	**0.63 (0.42‐0.94); 0.022**	**0.50 (0.31‐0.81); 0.005**	0.67 (0.45‐1.02); 0.060	**0.59 (0.40‐0.85); 0.005**
Smoking							
Yes	71/69	104/121	26/53	0.84 (0.55‐1.29); 0.426	**0.48 (0.27‐0.85); 0.012**	**0.53 (0.32‐0.88); 0.015**	0.73 (0.49‐1.09); 0.125
No	87/69	124/144	58/75	0.68 (0.46‐1.02); 0.060	**0.61 (0.39‐0.98); 0.040**	0.78 (0.53‐1.16); 0.216	**0.66 (0.45‐0.96); 0.028**
Alcohol							
Yes	76/62	111/124	45/53	0.68 (0.45‐1.04); 0.073	0.64 (0.38‐1.08); 0.093	0.81 (0.52‐1.27); 0.366	**0.67 (0.45‐0.99); 0.047**
No	82/76	117/141	39/75	0.83 (0.56‐1.24); 0.362	**0.52 (0.32‐0.86); 0.011**	**0.59 (0.38‐0.90); 0.015**	0.72 (0.49‐1.06); 0.092
Age (y)							
<60	97/80	123/157	54/72	**0.65 (0.45‐0.95); 0.026**	**0.62 (0.39‐0.98); 0.041**	0.80 (0.54‐1.20); 0.284	**0.64 (0.45‐0.91); 0.014**
≥60	61/58	105/108	30/56	0.92 (0.59‐1.45); 0.731	**0.51 (0.29‐0.90); 0.021**	**0.54 (0.33‐0.88); 0.013**	0.78 (0.51‐1.20); 0.259
BMI							
<25	74/66	119/139	45/61	0.77 (0.51‐1.16); 0.212	0.66 (0.40‐1.09); 0.107	0.78 (0.51‐1.20); 0.260	0.74 (0.50‐1.09); 0.123
≥25	84/72	109/126	39/67	0.74 (0.49‐1.11); 0.149	**0.50 (0.30‐0.83); 0.007**	**0.60 (0.38‐0.93); 0.022**	**0.66 (0.45‐0.96); 0.031**

Rs1799986	CC	CT	TT	CT vs. CC	TT vs.CC	TT vs. CT + CC	TT + CT vs.CC
Sex							
Male	123/127	79/94	11/26	0.86 (0.58‐1.27); 0.450	**0.43 (0.21‐0.92); 0.028**	**0.46 (0.22‐0.96); 0.038**	0.77 (0.53‐1.11); 0.162
Female	164/155	81/109	12/20	0.70 (0.49‐1.01); 0.056	0.57 (0.27‐1.20); 0.138	0.65 (0.31‐1.35); 0.246	**0.68 (0.48‐0.96); 0.030**
Smoking							
Yes	115/134	75/86	10/23	**0.63 (0.44‐0.89); 0.010**	**0.49 (0.24‐0.99); 0.048**	0.50 (0.3‐1.08); 0.078	0.90 (0.62‐1.32); 0.592
No	172/148	85/117	13/23	1.01 (0.68‐1.50); 0.966	0.50 (0.23‐1.10); 0.086	0.58 (0.29‐1.18); 0.132	**0.60 (0.43‐0.85); 0.003**
Alcohol							
Yes	148/128	78/94	12/17	0.71 (0.49‐1.04); 0.082	0.61 (0.28‐1.32); 0.206	0.69 (0.32‐1.48); 0.340	0.70 (0.48‐1.00); 0.052
No	139/154	82/109	11/29	0.83 (0.58‐1.20); 0.331	**0.42 (0.20‐0.87); 0.020**	**0.45 (0.22‐0.92); 0.030**	0.75 (0.53‐1.06); 0.101
Age (y)							
<60	174/164	88/121	13/24	**0.68 (0.48‐0.97); 0.031**	0.51 (0.25‐1.03); 0.060	0.59 (0.29‐1.18); 0.134	**0.65 (0.47‐0.91); 0.012**
≥60	113/118	72/82	10/22	0.92 (0.61‐1.38); 0.677	0.48 (0.22‐1.05); 0.065	0.49 (0.23‐1.07); 0.072	0.82 (0.56‐1.21); 0.326
BMI							
<25	142/136	84/111	12/19	0.72 (0.50‐1.04); 0.080	0.60 (0.28‐1.28); 0.188	0.69 (0.33‐1.45); 0.324	0.70 (0.49‐1.00); 0.050
≥25	145/146	76/92	11/27	0.83 (0.57‐1.22); 0.343	**0.41 (0.20‐0.86); 0.018**	**0.44 (0.21‐0.91); 0.026**	0.74 (0.51‐1.06); 0.095

Note

Bold values are statistically significant (*P* < .05).

**Table 6:** The associations between rs2908004/rs1799986 polymorphism and clinical characteristics of osteoarthritis

Characteristics	Genotype distributions			
Rs2908004	GG	GA	AA	GA + AA
ESR				
≥10/＜10	61/97	97/131	32/52	129/183
OR (95%CI); *P*‐value	1.0 (reference)	1.18 (0.78‐1.78); .439	0.98 (0.57‐1.69); .938	1.12 (0.76‐1.65); .568
CRP				
≥25/<25	15/143	10/218	3/81	13/299
OR (95%CI); *P*‐value	1.0 (reference)	0.44 (0.19‐1.00); .054	0.35 (0.10‐1.26); .095	**0.41 (0.19‐0.89); .021**
K‐L grade				
Ⅲ + Ⅳ/Ⅰ + Ⅱ	133/25	170/58	59/25	229/83
OR (95%CI); *P*‐value	1.0 (reference)	**0.55 (0.33‐0.93); .024**	**0.44 (0.24‐0.84); .011**	**0.52 (0.32‐0.85); .009**
VAS				
≥6/＜6	151/7	213/15	77/7	290/22
OR (95%CI); *P*‐value	1.0 (reference)	0.66 (0.26‐1.65); .371	0.51 (0.17‐1.51); .216	0.61 (0.26‐1.46); .265
Lequesnes’ index				
≥12/<12	153/5	218/10	81/3	299/13
OR (95%CI); *P*‐value	1.0 (reference)	0.71 (0.24‐2.13); .542	0.88 (0.21‐3.79); .866	0.75 (0.26‐2.15); .593

Rs1799986	CC	CT	TT	CT + TT
ESR				
≥10/＜10	111/176	70/90	8/15	78/105
OR (95%CI); *P*‐value	1.0 (reference)	1.23 (0.83‐1.83); .295	0.85 (0.35‐2.06); .712	1.18 (0.81‐1.72); .395
CRP				
≥25/<25	17/270	9/151	1/22	10/173
OR (95%CI); *P*‐value	1.0 (reference)	0.95 (0.41‐2.18); .897	0.72 (0.09‐5.68); 0.756	0.92 (0.41‐2.05); .835
K‐L grade				
Ⅲ + Ⅳ/Ⅰ + Ⅱ	250/37	135/25	20/3	155/28
OR (95%CI); *P*‐value	1.0 (reference)	0.80 (0.46‐1.38); .423	0.99 (0.28‐3.48); .983	0.82 (0.48‐1.39); .461
VAS				
≥6/<6	274/13	144/16	19/4	163/20
OR (95%CI); *P*‐value	1.0 (reference)	**0.43 (0.20‐0.91); .024**	**0.23 (0.07‐0.76); .009**	**0.39 (0.19‐0.80); .008**
Lequesnes’ index				
≥12/<12	277/10	152/8	22/1	174/9
OR (95%CI); *P*‐value	1.0 (reference)	0.69 (0.27‐1.77); .435	0.79 (0.10‐6.49); .829	0.70 (0.28‐1.75); .442

Note

Bold values are statistically significant (*P* < .05).

Weighted GRS revealed that individuals in the third/second versus the first quartile had a lower risk of developing OA (Table 7). This relationship remained true after adjusting for sex and age.

**Table 7:** The association of genetic risk score of Wnt signalling pathway‐related genes with risk of osteoarthritis

Variable	Case (n = 471)	Control (n = 532)	OR (95% CI)	*P* value	Adjusted OR (95% CI)	*P* value
Weighted GRS	N (%)	N (%)				
First (−0.5,0)	311 (66.9%)	287 (53.9%)	1		1	
Second (−1.0, −0.5)	149 (31.6%)	216 (40.6%)	**0.64 (0.49,0.83)**	**.001**	**0.63 (0.49,0.82)**	**.001**
Third (−1.5, −1.0)	5 (1.1%)	24 (4.5%)	**0.19 (0.07,0.51)**	**.001**	**0.19 (0.07,0.51)**	**.001**

Note

The genotyping was successful in 465 cases and 527 controls. Adjusted for sex and age. Bold values are statistically significant (*P* < .05).

**Figure 3:**
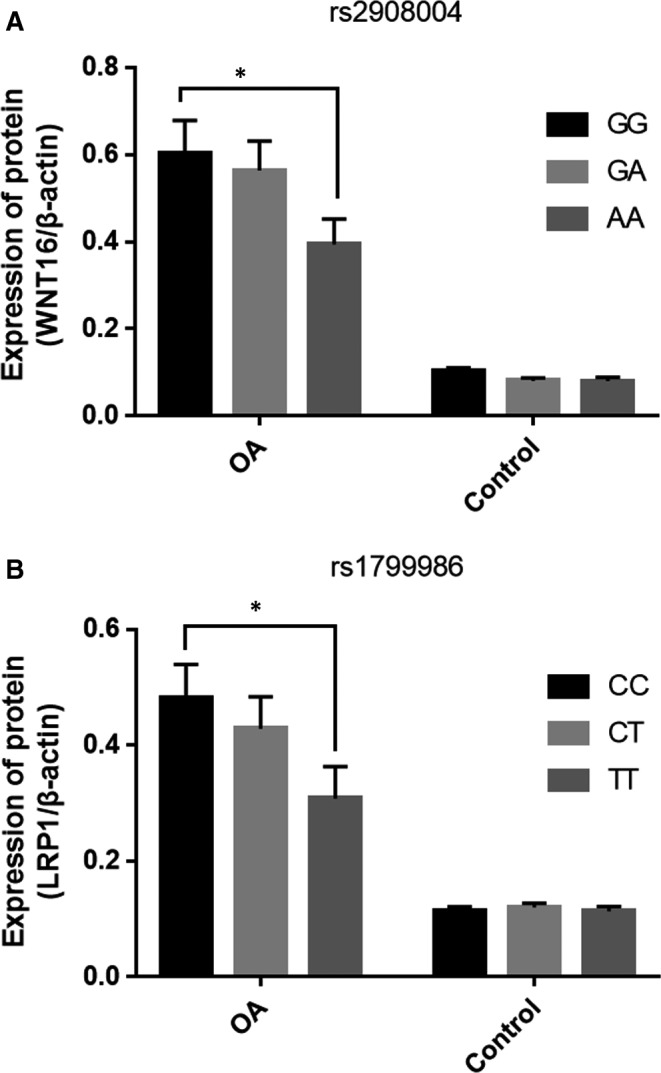
The mRNA expression by the genotypes of two polymorphisms among cases and controls. (A) WNT16 rs2908004 polymorphism; (B) LRP1 rs1799986 polymorphism

The mRNA expression of WNT16 or LRP1 was regulated by the genotypes of rs2908004 or rs1799986 polymorphism (Figure 3). Our results indicated that WNT16 and LRP1 were upregulated in OA compared to that in healthy controls. There existed a significant difference in the mRNA expression for rs2908004 or rs1799986 polymorphism (*P* < .05).

## 4 DISCUSSION

Two polymorphisms (rs2908004 and rs1799986) correlated with a decreased risk of OA, while no significant association was found between other polymorphisms (rs61735963, rs10795550 or rs1127379) and OA risk. Individuals with lower GRS had a lower risk of developing OA. In addition, rs2908004 or rs1799986 polymorphism reduces the expression of WNT16 or LRP1.

β‐catenin‐dependent canonical and independent non‐canonical Wnt signalling pathways play multiple roles in regulating the cartilage development, growth and maintenance in animal models.^15^ Alterations in genes associated with Wnt/β‐catenin signalling were suggested as susceptibility factors for OA development.^16^ Some SNPs of Wnt‐related genes influence the genetic predisposition to OA. Therefore, 13 studies changed their focus to the association of polymorphisms in pathway‐related genes with risk of OA. Careful reviewing suggests these 13 articles involved 20 genes and 127 SNPs. Twelve of the 127 SNPs were associated with OA among Caucasians. However, no study uncovered any significant association between these SNPs and OA risk among Asians.

As reported, WNT16 supported the homeostasis of progenitor cells in OA, while WNT16‐deficient mice developed more severe OA with lubricin downregulation and increased chondrocyte apoptosis.^17^ The expression of inter‐alpha inhibitor H5 (ITIH5) differed significantly between OA synovial tissues and healthy tissues.^18^ Lipoprotein receptor‐related protein 1 (LRP1) could inhibit tumour necrosis factor (TNF)‐α‐induced apoptosis and inflammation in chondrocytes.^19^ The reduced expression of secreted frizzled related protein 1 (SFRP1) increased the activation of the Wnt/β‐catenin signalling and rendered the articular cartilage prone to premature.^20^ We selected five SNPs (DVL1 rs61735963, WNT16 rs2908004, ITIH5 rs10795550, LRP1 rs1799986 and SFRP1 rs1127379) and investigated their association with OA risk in a Chinese population. Two SNPs (WNT16 rs2908004 and LRP1 rs1799986) were reported.^21^, ^22^ WNT16 could influence bone mineral density (BMD), cortical bone thickness and osteoporotic fracture risk.^23^ Radiographic OA was associated with high BMD and increased rate of bone loss.^24^ WNT16 rs2908004 polymorphism was associated with different OA phenotypes (atrophic or hypertrophic).^21^ Our results indicate WNT16 rs2908004 polymorphism conferred susceptibility to OA by comparing genotype and allele distribution between cases and controls. LRP1 dictates physiological and pathological catabolism of aggrecan by regulating extracellular activity of ADAMTS5.^25^ Inhibited shedding of low‐density LRP1 reversed the cartilage matrix degradation in OA.^26^ The role of LRP1 rs1799986 polymorphism was studied in various diseases, including cardiovascular disease 27 and Alzheimer's disease (AD).^22^ LRP1 rs1799986 polymorphism was associated with lower risk of AD among Asians and could reduce risk of late onset of AD, as revealed by a meta‐analysis containing 6455 AD cases and 6304 controls.^22^ However, little is known about their association with OA susceptibility. We found TT genotype or T allele significantly increased risk of OA in a Chinese population. Furthermore, the mRNA expression of WNT16 or LRP1 in mutant genotype carriers was significantly lower than that of wild genotype carriers. We hypothesized that rs2908004 or rs1799986 polymorphism conferred susceptibility to OA by influencing its production and function.

Several limitations of this study should be addressed. First, other confounders (eg occupation and diet) were not considered. Second, we recruited cases and controls only from hospitals, which may lead to selection bias during the collection process. Third, we may obtain the false‐negative or false‐positive results due to limited sample size. Fourth, two positive SNPs in exons may affect the protein expression, leading to OA susceptibility. However, this was not verified by experiments.

This study confirms that WNT16 rs2908004 polymorphism plays a protective role in the pathology of knee OA. Additionally, LRP1 rs1799986 polymorphism is associated with a decreased risk of OA. Nevertheless, larger‐size and well‐designed case‐control studies among other populations are warranted to identify the association between these polymorphisms and knee OA susceptibility.

## CONFLICT OF INTEREST

The authors declare that they have no conflict of interest.

## AUTHOR CONTRIBUTIONS

YH, XZ and LJ carried out the main experiments and statistical analysis and prepared the manuscript. HY and LW designed the study and prepared the manuscript. YH and XZ wrote the main protocol and prepared the manuscript. YH, JL and NX supervised the study and prepared the manuscript. All authors contributed to and have approved the final manuscript.
